# LncRNA LBX2-AS1 promotes proliferation and migratory capacity of clear cell renal cell carcinoma through mitophagy

**DOI:** 10.1186/s40001-024-01690-1

**Published:** 2024-02-07

**Authors:** Bao Wang, Yuang Wei, Tian Han, Peng Ji, Haoqi Miao, Xiangzheng Wu, Jian Qian, Pengfei Shao

**Affiliations:** Department of Urology, First Affiliated Hospital of Nanjing Medical University, Nanjing, China

**Keywords:** LncRNA LBX2-AS1, Mitophagy, Clear cell renal cell carcinoma

## Abstract

**Background:**

Long non-coding RNAs (lncRNAs) have been extensively investigated in the field of cancer, among which, lncRNA ladybird homeobox 2-antisense RNA 1 (LBX2-AS1) has been demonstrated to exert carcinogenic effects on a variety of malignancies. However, the biological functions of LBX2-AS1 in clear cell renal cell carcinoma (ccRCC) have not been explicitly elucidated.

**Methods:**

Arraystar lncRNA chip and qRT-PCR verify the expression of LncRNA LBX2-AS1 in ccRCC. CCK-8 assay and cell cloning assay were used to assess the proliferative capacity of ccRCC cells. Migration abilities were quantified by scratch assay and transwell assay. Potential molecular signaling pathways were determined by high-throughput whole transcriptomics analysis. WB analysis was performed to validate the relationship between LBX2-AS1 and key molecules of mitophagy pathway. The effect of LBX2-AS1 on mitophagy was observed by laser confocal microscopy. Rescue experiments further validated the role of downstream gene FOXO3A in the LBX2-AS1 signaling pathway. Finally, the authentic effect of LBX2-AS1 was verified in vivo.

**Results:**

LncRNA LBX2-AS1 was over expressed in ccRCC tissues and could enhance the proliferation and migration of ccRCC cells. Autophagic pathway was identified as a possible mechanism involved in the oncogenic effect of LBX2-AS1. Mitophagy levels were observed in LBX2-AS1 low-expressing cells through laser confocal microscopy. Knockdown of LBX2-AS1 significantly elevated mitophagy levels as observed using laser confocal microscopy and led to FOXOA3 decreasing in and BNIP3L and LC3 enrichment. Meanwhile, LBX2-AS1 knocking down dampened the proliferation of ccRCC cells in vivo.

**Supplementary Information:**

The online version contains supplementary material available at 10.1186/s40001-024-01690-1.

## Introduction

Renal cell carcinoma, as the most lethal malignancy of the urinary tract with multiple subtypes, has been the subject of extensive research. Among its diverse subtypes, clear cell renal cell carcinoma (ccRCC) has garnered particular attention in investigations into its developmental mechanisms.

While protein-coding genes have traditionally been focal points in physiopathological processes, it is irrefutable that the non-protein-coding elements of the human genome constitute a considerable portion and exert a crucial role in regulating gene stability and biological function [1]. Two categories of non-coding RNAs are classified based on the length. Those less than 200 nucleotides in length are called small non-coding RNAs, while long non-coding RNAs (lncRNAs) are regarded as having more than 200 nucleotides in length [2]. It is estimated that the human genome encodes at least 20,000 lncRNAs, which play a key role in regulating gene expression and are involved in various cellular signaling [3]. A growing body of evidence suggests that lncRNAs are major participants in cancer fields, attributed to the fact that lncRNAs are involved in a wide range of cellular mechanisms, including sophisticated interactions with RNA, DNA, and proteins [4]. Therefore, lncRNAs have a promising prospect as the biomarkers and therapeutic targets for malignant tumors.

Ladybird homeobox2 (LBX2)-antisense RNA 1 (AS1) is a recently identified non-coding RNA that plays an important biological function in numerous tumor and non-tumor disorders. Subcellular localization analysis indicated that LBX2-AS1 was mainly available in the cytoplasm and less distributed in the nucleus. Available studies suggest that LBX2-AS1 exerts a promotive effect in tumors such as glioma, colorectal cancer, osteosarcoma, ovarian cancer, hepatocellular carcinoma, esophageal squamous cell carcinoma, and thyroid cancer. Studies on the LBX2-AS1-associated ceRNA network have shown that LBX2-AS1 could enhance tumor cell proliferation and migration by sponging numerous downstream miRNAs. For example, LBX2-AS1 was observed to suppress miR-491-5p function in ovarian cancer, colorectal cancer, gastric cancer, and glioma [5–8], thereby upregulating oncogenes and mediating the malignant phenotypes. It has also been shown to affect apoptosis of tumor cells in glioma and hepatocellular carcinoma [6]. Nonetheless, the exploration of LBX2-AS1 in ccRCC remains largely uncharted. Hence, the aim of this study is to elucidate the biological function and potential underlying mechanisms of LBX2-AS1 in the context of ccRCC.

## Methods

### Cell culture, lentiviral shRNA transfection and plasmid vector transfection

Human ccRCC cell lines 769-P and Caki-1 were purchased from the Chinese Academy of Sciences Cell Bank (Shanghai, China). 769-P was cultured in 1640 medium containing 10% fetal bovine serum and Caki-1 was cultured in McCoy’s 5A medium containing 10% fetal bovine serum. Lentiviral vector-based small hairpin RNA (shRNA) targeting LBX2-AS1 was ordered from GenePharma (Shanghai, China). Plasmid vector-based DNA to overexpress FOXO3A was purchased from GeneChem (Shanghai, China). For stable knockdown of LBX2-AS1 in cells, ccRCC cell lines were transfected with lentivirus for 24 h and filtered by puromycin killing effects for 2 to 3 times. For stable overexpression of FOXO3A in cells, ccRCC cell lines were transfected with plasmids using Lipofectamine 3000 (Thermo Fisher, United States) for 4 to 6 h. The efficiency of genetic interference was confirmed by qRT-PCR or Western blotting.

### Quantitative real-time polymerase chain reaction (qRT-PCR)

The RNA Isolation Kit (Vazyme, R333, Nanjing) was used to extract total RNA from cells and tissues. qPCR was performed after reverse transcription using SYBR Green Master Kit as the fluorescent dye, and the StepOne™ Real-Time Quantitative Fluorescence PCR Instrument (Thermo Fisher Scientific, Rochester, NY, United States) was employed to amplify the DNA. Each qRT-PCR was performed in triplicate and β-actin was used for normalization of gene expression. Primer sequences for gene amplification are listed in Additional file 1: Table S1.

### Western blot

RIPA lysate was used for cell lysis and protein extraction, and BCA was used to determine the protein concentration of the samples for western blotting. Proteins were separated by 8% sodium dodecyl sulfate–polyacrylamide gel electrophoresis (SDS-PAGE) and then transferred to polyvinylidene difluoride (PVDF) membranes. The PVDF membranes were closed in 5% skimmed milk for 3 h at room temperature and incubated overnight at 4 °C with specific primary antibodies. On the second day, the membranes were incubated with anti-mouse or anti-rabbit IgG secondary antibodies for 2 h at room temperature. After washing the PVDF membranes with TBST, protein imprinting was visualized using an enhanced chemiluminescence (ECL) detection system (Thermo Fisher Scientific, Rochester, NY, United States).

### Loss-of-function experiments and rescue experiments

The CCK-8 assay and colony formation assay were used to assess proliferative capacity of cells. A total of 10^3^ cells from shLBX2-AS1 group and shNC group were spread into the 96-well plates and cultured in an environment of 5% CO_2_ and 37 °C, and the absorbance at OD 450 nm of each well was measured by multifunctional enzyme marker at 24, 48, 72 and 96 h, respectively. Meantime, a total of 10^3^ cells of shLBX2-AS1 group and shNC group were spread into 6-well plates, respectively, and the cells were stained with crystal violet solution for one week. The number of clonal community formation was counted.

The migration assay and scratch assay were used to assess the migration ability of cells. A serum-free medium suspension containing 2 × 10^4^ cells was placed in the upper layer of the chamber, and the lower layer was cell culture medium containing 10% fetal bovine serum. After 36 to 48 h of migration, tumor cells in the upper layer were washed away and the number of cells in the lower layer was stained with crystal violet solution and recorded under a light microscope.

### Subcutaneous tumor formation in nude mice

A suspension of 769-P cells and Caki-1 cells from the shLBX2-AS1 and NC groups was injected subcutaneously into nude mice. After the tumor is formatted, the volume of subcutaneous tumors was measured every 3 days during the period. 25 days later, the nude mice were executed and the subcutaneous tumors were extracted for following analysis.

## Results

### LBX2-AS1 is highly expressed in ccRCC both in situ and in vitro

**Fig. 1:**
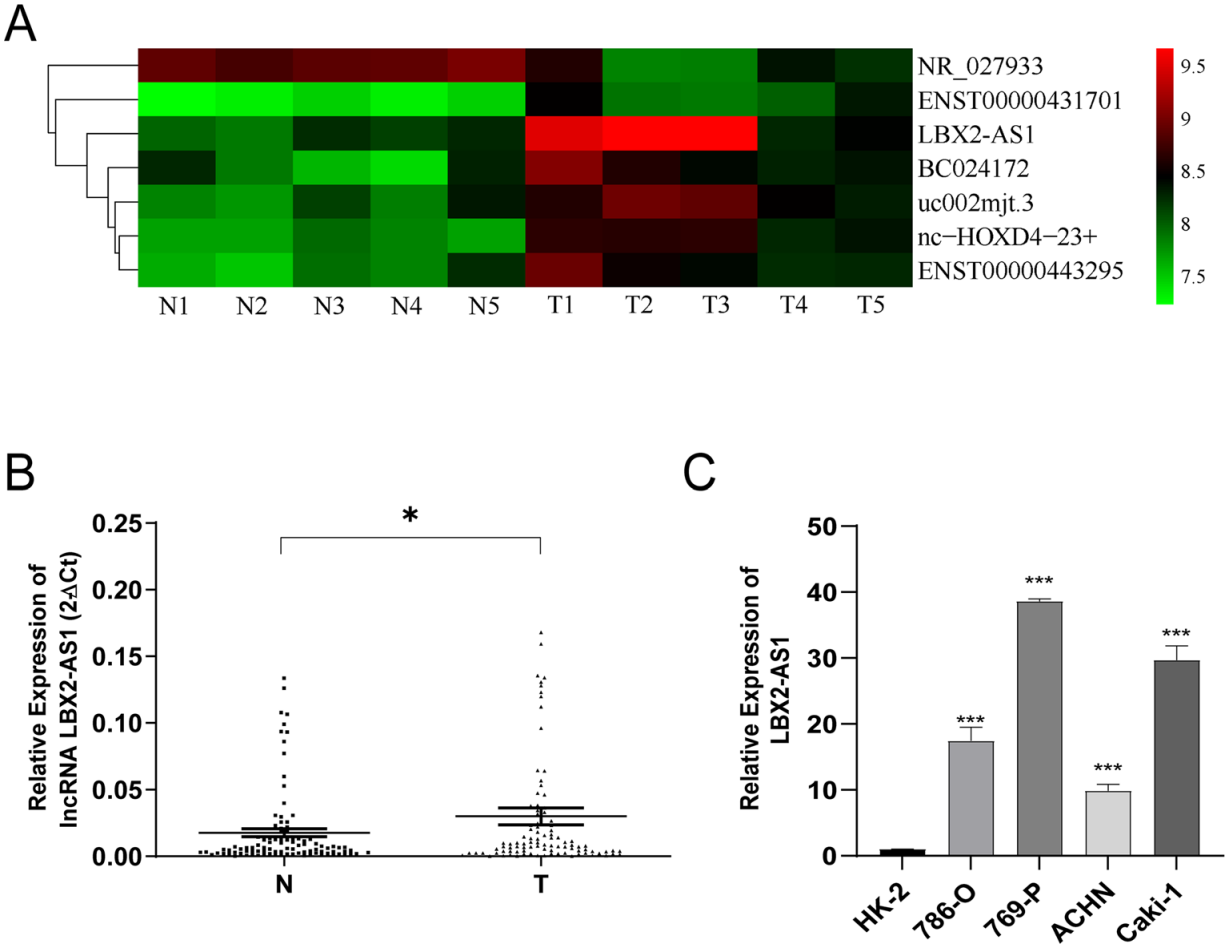
Elevated expression of LBX2-AS1 in ccRCC. **A** The heatmap of lncRNAs with aberrant expression between tumor and normal tissues of ccRCC. **B** The expression of LBX2-AS1 in 96 pairs of ccRCC tissues by qPCR. **C** The expression of LBX2-AS1 in ccRCC cell lines by qPCR. *P < 0.05, ***P < 0.001

To clarify the expression profile of LncRNAs in ccRCC, the tumor and adjacent non-tumor tissues of five ccRCC patients were analyzed using Arraystar lncRNA microarrays. LBX2-AS1 was found to exhibit significantly higher expression in ccRCC tissues compared to adjacent tissues (Fig. 1A). The overexpression of LBX2-AS1 was subsequently validated in 96 paired ccRCC tissues and adjacent non-tumor tissues using qRT-PCR (Fig. 1B). Consistent results were observed in various renal cancer cell lines, including 786-O, 769-P, ACHN, and Caki-1 (Fig. 1C). Consequently, the upregulation of LBX2-AS1 in cancer was confirmed, and 769-P and Caki-1 were selected for further investigation based on their relatively higher expression levels.

### Knockdown of LBX2-AS1 significantly impaired the proliferation and migration ability of ccRCC cells

**Fig. 2:**
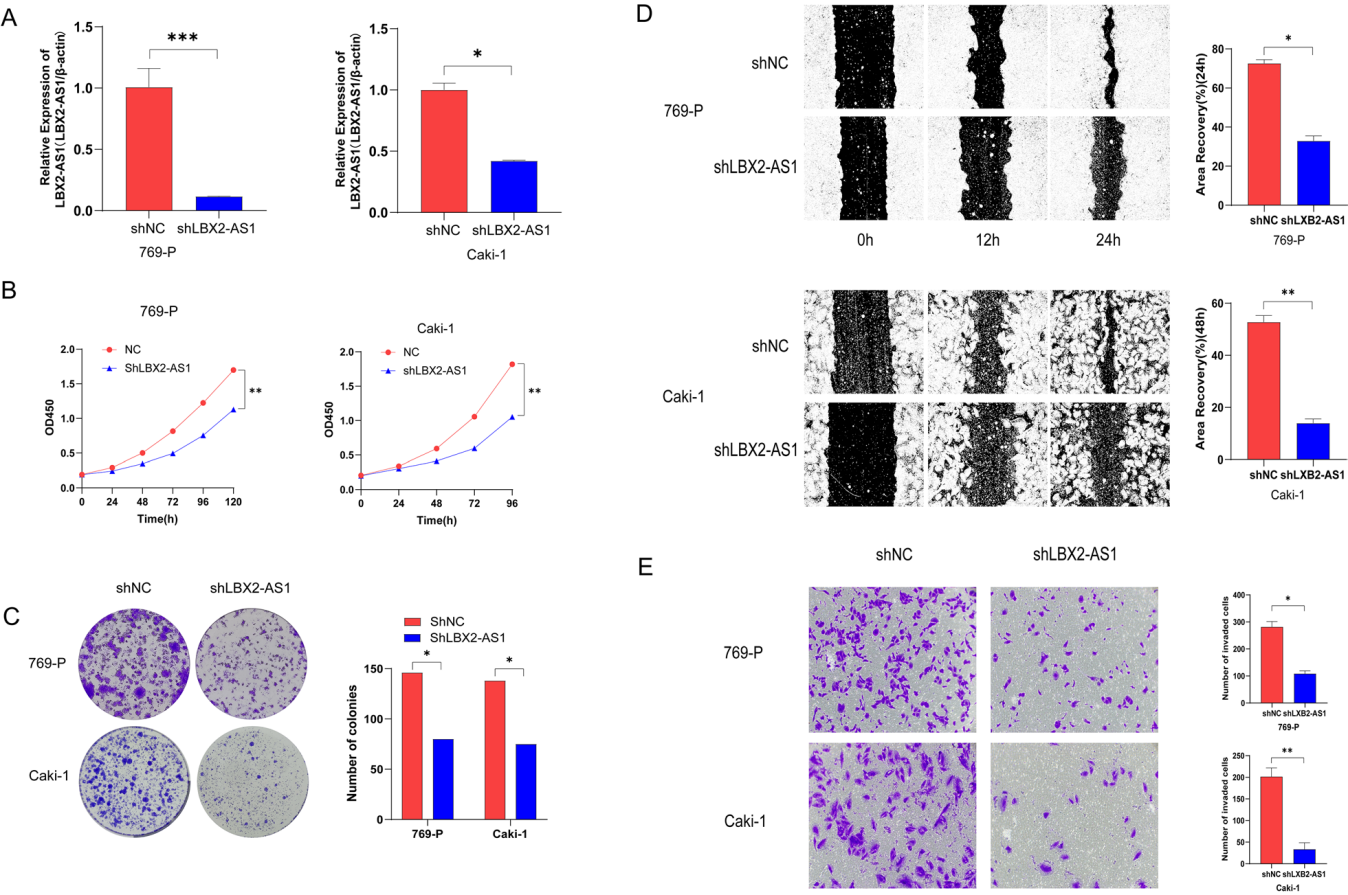
LBX2-AS1 promoted the proliferation and migration ability of ccRCC cells. **A** The validation of LBX2-AS1 knockdown by qPCR. **B**–**E** The results of CCK-8 assay (**B**), colony formation assay (**C**), scratch assay (**D**) and transwell assay (**E**) in shLBX2-AS1 and shNC groups. *P < 0.05, **P < 0.01, ***P < 0.001

Using the prescribed methodology, lentivirus-transfected cell lines targeting LBX2-AS1 (shLBX2-AS1) and a negative control (shNC) were successfully generated (Fig. 2A). Evaluation through CCK-8 and colony formation assays demonstrated a notable inhibition in the proliferation capacity of 769-P and Caki-1 cells upon LBX2-AS1 knockdown (Fig. 2B, C). Furthermore, the scratch and transwell assays indicated that reduced levels of LBX2-AS1 could effectively suppress the migration ability of ccRCC cells (Fig. 2D, E).

### LBX2-AS1 could promote the malignant phenotype of ccRCC by regulating mitochondrial autophagy

**Fig. 3:**
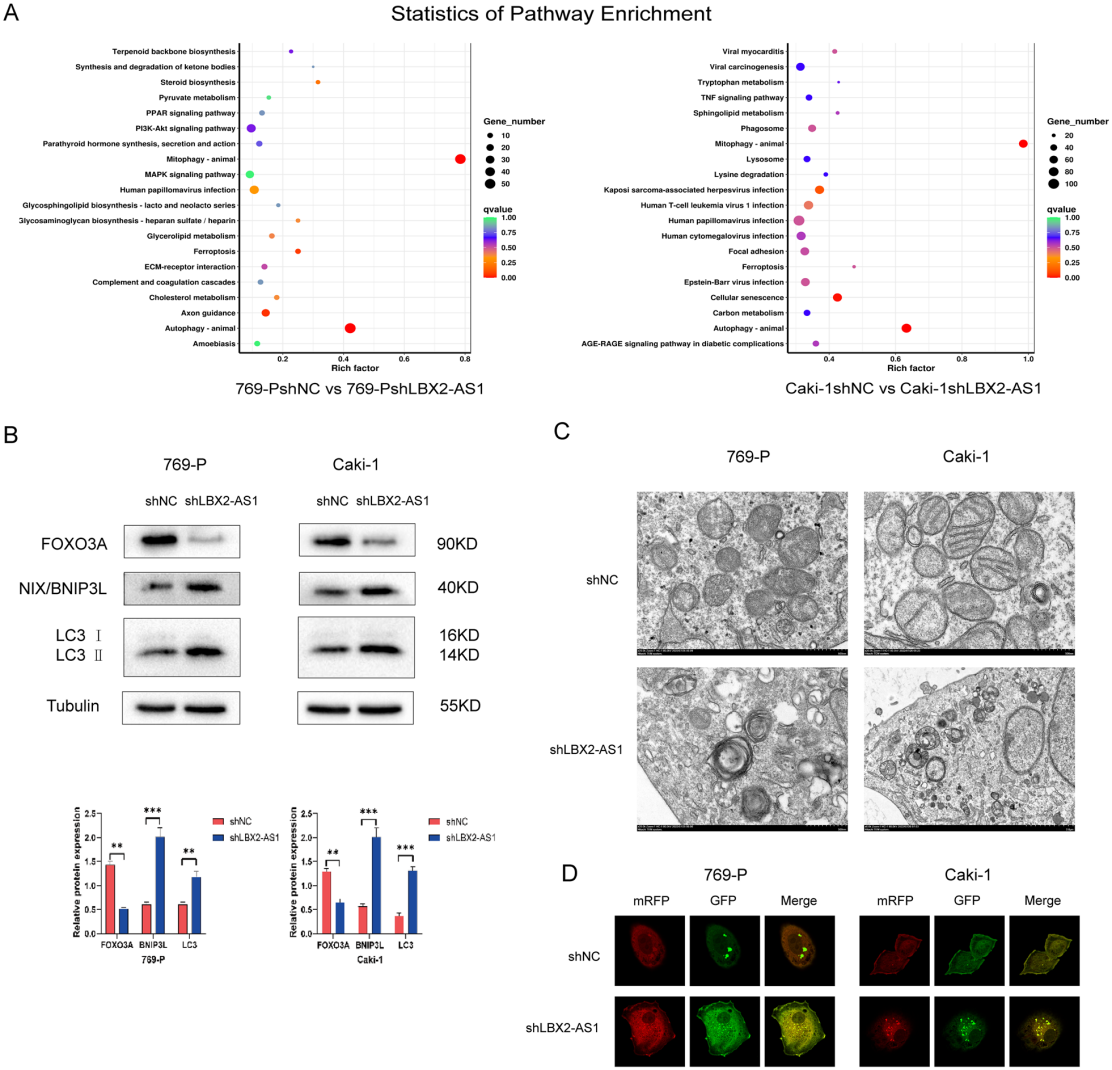
Mitophagy-related role of LBX2-AS1 in regulating tumor progression. **A** The results of KEGG analysis on shLBX2-AS1 cells. **B** The protein level alteration of autophagy pathway in shLBX2-AS1 cells. **C** The results of morphologic changes in shLBX2-AS1 cells under TEM. **D** The results of morphologic changes in shLBX2-AS1 cells under LCM

To reveal the mechanism of the oncogenic potential of LBX2-AS1, RNA-seq analysis was conducted on shLBX2-AS1 cell lines. The findings revealed a prominent enrichment of the mitophagy pathway in both 769-P and Caki-1 cells (Fig. 3A), suggesting a potential association between the phenotypic effects of LBX2-AS1 and the mitophagy pathway. Subsequently, alterations in the mitochondria pathway were assessed. Western blot analysis indicated a significant decrease in FOXO3A protein levels, along with upregulated expression of NIX/BNIP3L and LC3 in the shLBX2-AS1 groups (*P* < 0.01, Fig. 3B). Additionally, transmission electron microscopy revealed evident mitochondrial damage and the presence of autophagic vesicles in cells from the shLBX2-AS1 group (Fig. 3C). Laser confocal microscopy further demonstrated distorted mitochondrial structures and the formation of autophagic vesicles upon shLBX2-AS1 intervention, indicating the occurrence of autophagy in mitochondria (Fig. 3D).

**Fig. 4:**
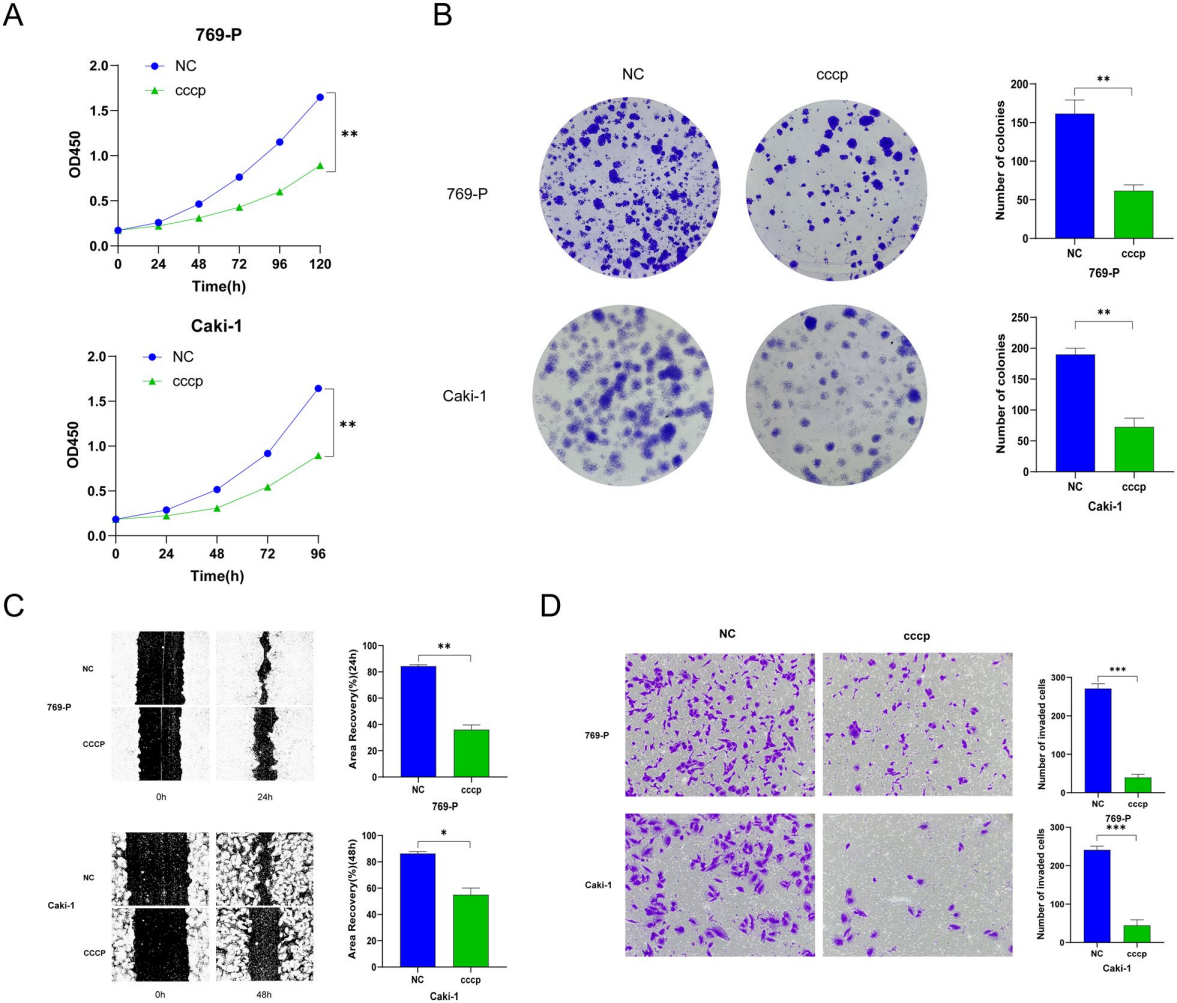
Mitochondrial autophagy of 769-P and Caki-1 was induced by CCCP, which significantly reduced their proliferation, migration and invasion. **A** CCK-8 experiment showed that increased mitochondrial autophagy inhibited the proliferation of 769-P and Caki-1 cells (**P < 0.01). **B** The results of cell cloning in vitro showed that the colony formation of renal carcinoma cells with low expression of LBX2-AS1 was inhibited (**P < 0.01). **C** Cell scratch test results showed that mitochondrial autophagy induced by CCCP in 769-P and Caki-1 decreased cell migration (*P < 0.05, **P < 0.01). **D** Transwell assay showed that the invasion ability of 769-P and Caki-1 cells could be inhibited after LBX2-AS1 expression was down-regulated (***P < 0.001)

Furthermore, investigations were conducted to assess the impact of mitophagy occurrence in ccRCC cells on malignant phenotypes. Carbonyl cyanide m-chlorophenylhydrazone (cccp) was employed to induce mitophagy in 769-P and Caki-1 cells. The results of CCK-8 and colony formation experiments indicated a significant inhibition of the proliferation ability of 769-P and Caki-1 upon cccp intervention (Fig. 4A, B). Similarly, scratch and transwell assays suggested a marked reduction in the migration ability of ccRCC cells following the induction of cccp (Fig. 4C, D).

### LBX2-AS1 restrained mitophagy in ccRCC cells via FOXO3A–BNIP3L–LC3 axis

**Fig. 5:**
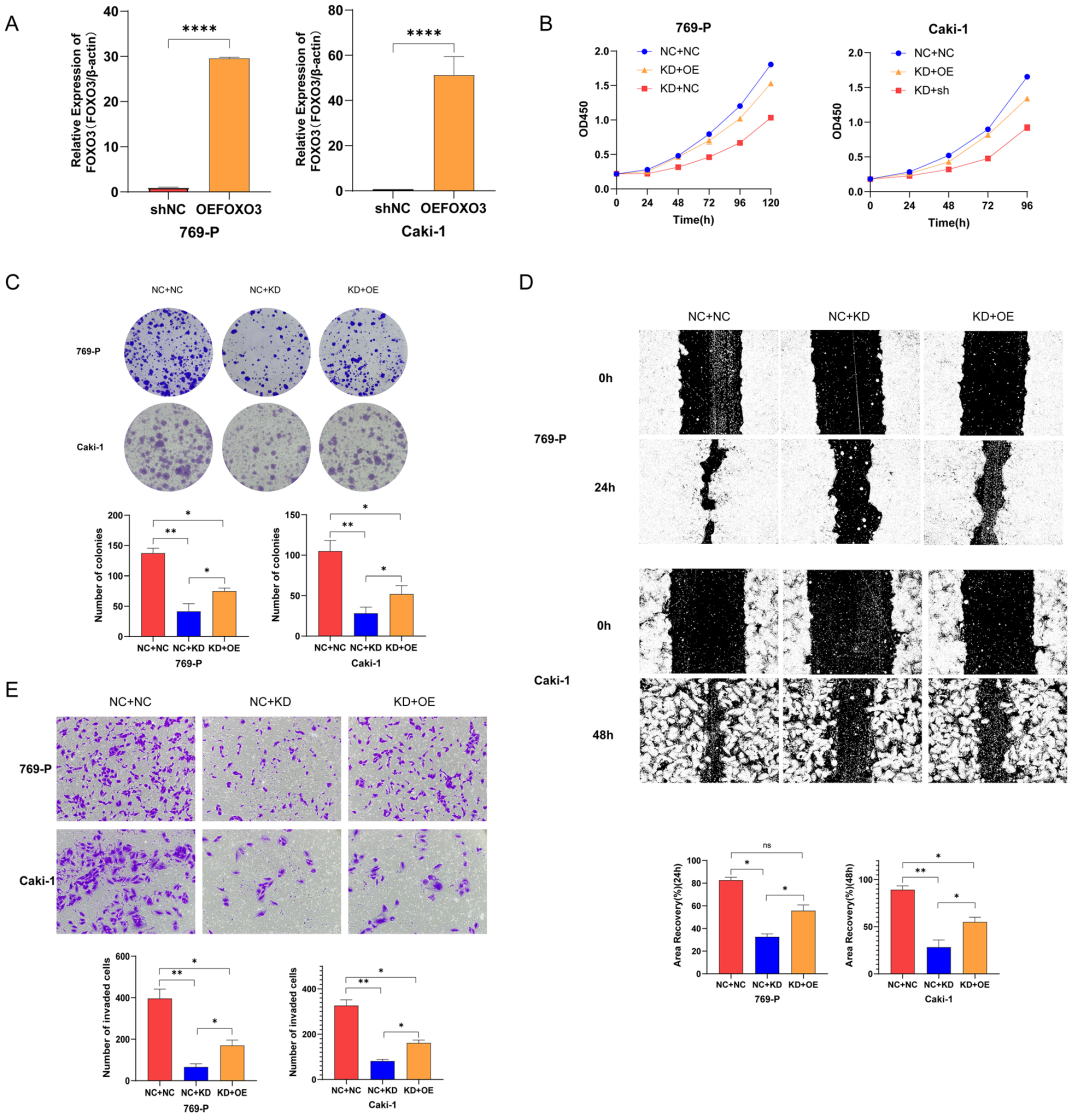
After knocking down the expression of LBX2-AS1 in renal carcinoma cells, overexpression of FOXO3 can partially restore the proliferation, invasion and migration of renal clear cell carcinoma. **A** RT-qPCR verified the efficiency of overexpression of FOXO3 by lentivirus. (****P < 0.0001). **B**, **C** The results of CCK8 and cell cloning tests showed that overexpression of FOXO3 cocoa partially restored the proliferation ability of renal clear cell carcinoma after low expression of LBX2-AS1 (*P < 0.05, **P < 0.01). **D**, **E** Scratch test and transwell test showed that overexpression of FOXO3 cocoa partially restored the migration and invasion ability of renal clear cell carcinoma after low expression of LBX2-AS1 (*P < 0.05, **P < 0.01)

Next, we conducted a screening to identify key genes in the mitophagy pathway. As a result of significant expression changes, FOXO3 and BNIP3L were determined as candidate genes. Based on KEGG analysis, we formulated a hypothesis suggesting that LBX2-AS1 regulates mitophagy through the FOXO3A–BNIP3L–LC3 axis. In rescue experiments, lentivirus-mediated transfection was used to achieve FOXO3A overexpression in 769-P and Caki-1 cells, and the efficiency was confirmed by RT-qPCR (Fig. 5A). Subsequently, FOXO3 was overexpressed in LBX2-AS1 knockdown cells. Evaluation through CCK-8 and cell cloning experiments revealed that overexpression of FOXO3 significantly mitigated the decline in cell proliferation caused by LBX2-AS1 knockdown (Fig. 5B, C). Furthermore, scratch and transwell tests demonstrated that FOXO3 overexpression enhanced cell migration and invasion abilities (Fig. 5D, E). These findings provide objective evidence supporting the hypothesis that LBX2-AS1 modulates mitophagy through the regulation of the FOXO3A–BNIP3L–LC3 axis. They shed light on the potential role of this pathway in the development and progression of ccRCC.

### In vivo experiments showed that knockdown of LBX2-AS1 inhibited subcutaneous tumor formation in mice

**Fig. 6:**
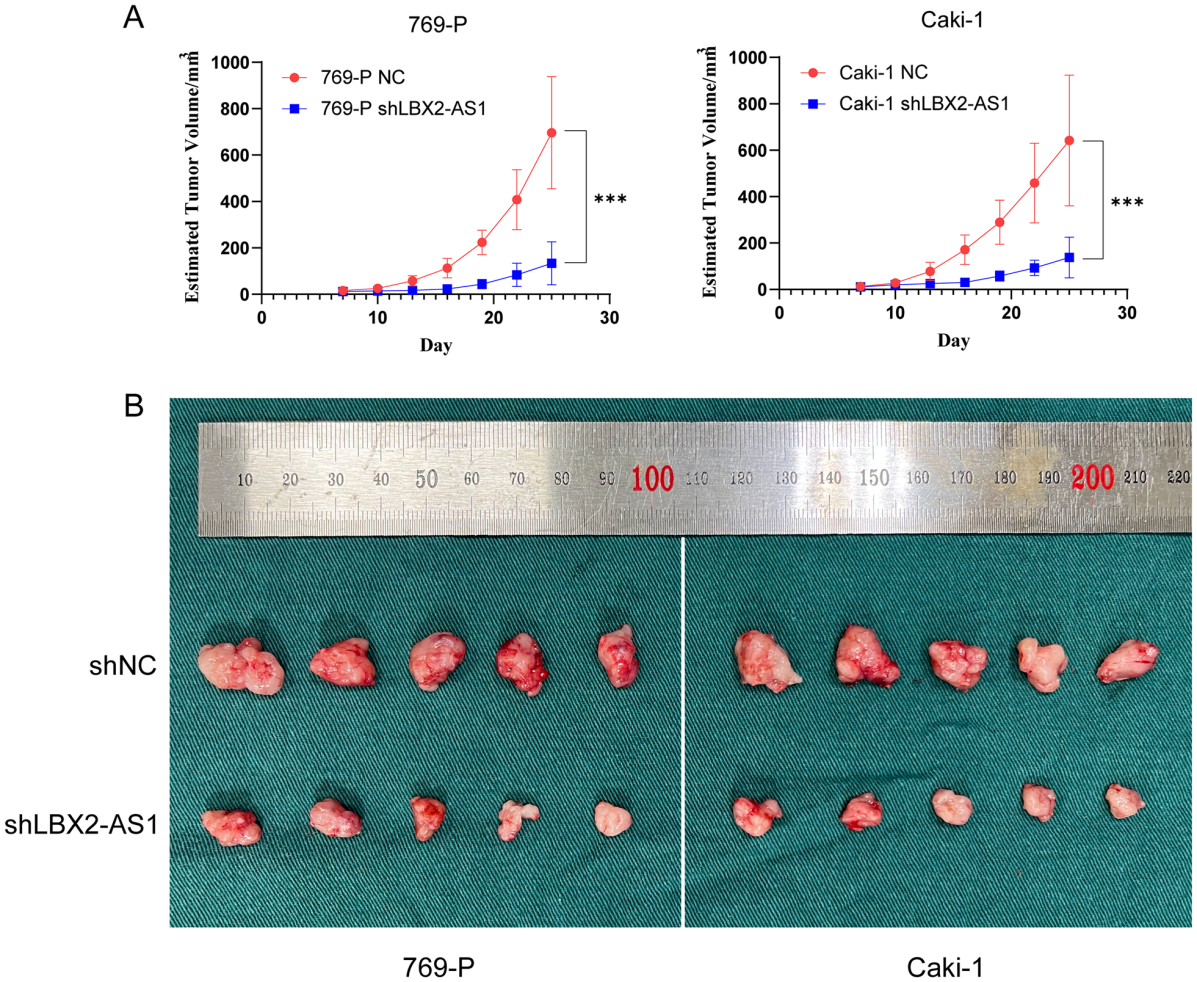
LBX2-AS1 promoted the tumor growth in vivo. **A** The growth curves of tumors of mice in shLBX2-AS1 and shNC groups. **B** The display of tumor formation and size. ***P < 0.001

Finally, subcutaneous tumor formation experiments were performed in nude mice to assess the tumorigenic capacity. The growth curves of ccRCC tumors reflected the distinction of tumor volume between shLBX2-AS1 group and shNC group, indicating the decreased tumor formation capability of cells resulted by knockdown of LBX2-AS1 (Fig. 6A, B).

## Discussion

The present study focused on the oncogenic effects and autophagy-related role of LBX2-AS1 in ccRCC. In our study, LBX2-AS1 was chosen for research based on the microarray results of 5 pairs of ccRCC samples. Among all differentially expressed lncRNAs, LBX2-AS1 demonstrated the most apparent upregulation in tumoral tissues. Despite of the relatively few numbers of samples subject to Arraystar analysis, the subsequent clinical validation of 96 pairs of tissues solidly confirmed the upregulation of LBX2-AS1 in ccRCC.

The early diagnosis of RCC is a persistent challenge in clinical management, and current tomography technologies have not fully met the requirements for identifying small renal masses and cystic lesions in a reliable manner [9]. Emerging approaches such as molecular biomarker, genomics, and artificial intelligence application in imaging [10, 11], have been considered promising in early diagnosis of urinary tumors. Etiologically, the activation of tumor microenvironment could be the driving force of tumorigenesis in these contexts [12]. The present study showed the upregulated LBX2-AS1 resulted in enhanced proliferation and migration capabilities of tumor cells. The LBX2-AS1 silence also inhibited the in vivo tumor formation of mice. Through the administration of transcriptomics, we further discovered the strong involvement of LBX2-AS1 in mitochondrial autophagy pathway. Furthermore, abundant laboratory results confirmed the suppressive role of LBX2-AS1 on mitophagy which benefited the tumor progression of ccRCC. Normally the primary way of LBX2-AS1 exerting oncogenic role in cancers is based on the mechanism of competing endogenous RNA (ceRNA) network. Specifically, LBX2-AS1 could counteract the negative effects of miRNA on mRNA translation, which is favor to the expression and stabilization of key factors in cancer-related signaling and vital regulatory pathways [13]. The classical functioning pattern has been demonstrated in numerous cancers [5–8, 14]. Previous studies have confirmed that LBX2-AS1 was an influential regulatory factor in cell proliferation, apoptosis, invasion and migration in tumors. Nevertheless, its role in regulating autophagy and related tumor progression has been rarely implicated. Meng et al. screened for autophagy-associated lncRNAs and found that LBX2-AS1 was an independent prognostic factor in ovarian cancer [15]. In the present study, our results confirmed that knockdown of LBX2-AS1 activated the mitophagy and observed the robust evidence of the formation of autophagic vesicles in cells. This finding could be considered as the first validation of autophagy-related role of LBX2-AS1 in cancers.

Autophagy is an adaptation mechanism that cells use in response to stress and protect themselves, and forkhead box O3 (FOXO3) A and BNIP3-like (BNIP3L)/NIX were determined as inductive factors of autophagy. As a transcription factor, FOXO3/FOXO3A promotes autophagy by transactivating related genes and, meantime, is itself degraded by autophagic regulation [16]. The dominant inductive effects of FOXO3A on autophagy makes the regulation of itself an effective way of deciding the cell fate. It has been widely proved that many upstream factors could regulate FOXO3A expression in different ways, such as Akt, SIRT1/3, and mTOR [17–20]. Results provided here suggested that LBX2-AS1 positively regulated the FOXO3A expression while negatively affected the BNIP3L level, and that knockdown of LBX2-AS1 apparently upregulated the mitophagy level through BNIP3L which finally suppressed the tumor progression. The positive regulatory role of LBX2-AS1 on FOXO3A was consistent with similar studies that reported the relationship between lncRNAs and FOXO3A. Hong et al. reported that by using RNA-seq, they found a coincident expression changes of lncRNA-SNHG14, miR-223-3p, and FOXO3A. Further experiments confirmed the binding relationship between SNHG14 and miR-223-3p, as well as the silencing effect of miR-223-3p on the FOXO3A expression [21]. Liang et al. found that lncRNA RP11-295G20.2 could regulate hepatocellular carcinoma cell growth by influencing Akt phosphorylation and FOXO3A translocation into the nucleus, which regulated the transcription of autophagy-related genes [22]. Likewise, RBM47/SNH5G signaling was found to stabilize FOXO3A and promote its translocation into nucleus, which activated autophagy and inhibited the cell proliferation of papillary thyroid carcinoma (PTC) [23]. Nevertheless, further exploration in our research found that in ccRCC, higher expression of FOXO3A restrained the mitophagy and enhanced the proliferation and migration capabilities of tumor cells, which was contrary to the outcome of previous studies. The likely explanation here could be that FOXO3A was involved in numerous signaling pathway and biological processes, which could benefit the progression of ccRCC [24, 25].

The other novel finding was that LBX2-AS1 negatively regulated BNIP3L expression and affected the autophagy activity in ccRCC. BNIP3L/NIX, known as BNIP3-like or NIP3-like protein X, was considered as the vital mitophagy receptor and executor [26]. BNIP3L/NIX is the outer mitochondrial membrane protein, and its expression increases during red blood cell differentiation which is required for mitochondrial removal. BNIP3L/NIX functions by binding to MAP1 light chain 3 (also known as LC3) on isolation membranes and mediate the binding and separation of mitochondria and autophagosomes [27]. Numerous studies showed that BNIP3L/NIX was capable of protecting cells from damaged mitochondria through inducing mitophagy in kinds of disease, including ischemic brains, lipotoxicity, synapse defects, cancers, and immunity and autoimmune diseases [26, 28–31]. Although BNIP3, not BNIP3L, was proved as the direct downstream target of FOXO3A in autophagy signaling, the interaction between FOXO3A and BNIP3L is still unclear and unreported [32]. Therefore, it was implicated in the present study that LBX2-AS1 knockdown led to the paradoxical influences of FOXO3A and BNIP3L on mitophagy level independently, and it finally manifested as upregulated mitochondrial autophagy in cells.

Still, the research is short of mechanistic explanation on the regulation of LBX2-AS1 on FOXOA1 and BNIP3L/NIX, and the potential mechanism of FOXO3A downregulating mitophagy and protecting tumor cells was not completely elaborated. These items are expected to be further investigated in future work.

### Supplementary Information

**Additional file 1.** **Table S1**. Sequences of primers used in qPCR.

## Data Availability

The original contributions presented in the study are included in the article/additional files. Further inquiries can be directed to the corresponding authors.
