# Supplementary material for: LncRNA LBX2-AS1 promotes proliferation and migratory capacity of clear cell renal cell carcinoma through mitophagy
Source: Eur J Med Res. 2024 Feb 7;29:103. doi: 10.1186/s40001-024-01690-1 (PMC10848470; doi:10.1186/s40001-024-01690-1)
Supplement: Supplementary file 1 — Additional file 1. Table S1. Sequences of primers used in qPCR. [file 40001_2024_1690_MOESM1_ESM.docx]

**Table S1**

| Primers | Sequences |
| --- | --- |
| LBX2-AS1 | F- TGCGCCTCCATACAGTTTGT  R- GAGGAGAGCAAGGGCAACTT |
| FOXO3 | F- ACAGCACCGAGGAGAACT  R- ATTGTCCATGGAGACAGCCC |
| β-actin | F- ACTGGAACGGTGAAGGTGAC  R- AGAGAAGTGGGGTGGCTTTT |
